# The incidence of cardiac complications in patients hospitalised with COVID‐19 in Australia: the AUS‐COVID study

**DOI:** 10.5694/mja2.51225

**Published:** 2021-08-17

**Authors:** Kunwardeep S Bhatia, William van Gaal, Leonard Kritharides, Clara K Chow, Ravinay Bhindi, the AUS‐COVID Investigators, Usaid Allahwala, Justin Chia, Christopher YP Choong, Karina Chui, Jonathan Ciofani, Anthony Delaney, Benjamin Harris, Bernard Hudson, Logan Kanagaratnam, George Kotsiou, Daniel Nour, Hari Prakash Sritharan, Rohan Bhagwandeen, David B Brieger, Andy Yong, Girish Dwivedi, Graham Hillis, Dhanvee Kandadai, George Javorsky, Nigel Jepson, Astin Lee, Sidney TH Lo, Andrew I MacIsaac, Brendan M McQuillan, Isuru Ranasinghe, Sheran Vasanthakumar, Antony Walton, James Weaver, Pavithra Jayadeva, William Wilson, John Zhu

**Affiliations:** ^1^ Royal North Shore Hospital Sydney NSW; ^2^ Northern Health Melbourne VIC; ^3^ Concord Repatriation General Hospital Sydney NSW; ^4^ ANZAC Research Institute Sydney NSW; ^5^ Westmead Clinical School University of Sydney Sydney NSW; ^6^ Westmead Hospital Sydney NSW; ^7^ Northern Clinical School University of Sydney Sydney NSW

**Keywords:** COVID‐19, Atrial fibrillation, Cardiomyopathies

Recognised cardiac complications of coronavirus disease 2019 (COVID‐19) include arrhythmias,^1^, ^2^ myopericarditis,^3^ and cardiomyopathy,^4^, ^5^ but data on their incidence are limited. The Australian Cardiovascular COVID‐19 Registry (AUS‐COVID), an observational cohort study in 21 hospitals, is assessing the incidence of cardiac complications of COVID‐19. The registry includes data on all index hospitalisations of people with laboratory‐confirmed severe acute respiratory syndrome coronavirus 2 (SARS‐CoV‐2) infections (protocol and case report form: https://www.aus‐covid.com). The registry was approved by the Northern Sydney Local Health District Human Research Ethics Committee (HREC2020/ETH00732), which granted a waiver of individual patient consent, and was prospectively registered with the Australian and New Zealand Clinical Trials Registry on 17 April 2020 (ACTRN12620000486921).

We analysed data for 644 consecutive patients included in the AUS‐COVID Registry by 28 January 2021 (mean age, 62.5 years; standard deviation, 20.1 years), including 329 men (51%). One hundred and twenty‐five patients (19%) were admitted to intensive care units, and 70 patients (11%) required intubation; 92 patients (14%) died in hospital. Outcomes for 15 patients (2%) transferred to other hospitals were not known.

Twenty of 553 patients without histories of atrial fibrillation or flutter were diagnosed with the condition (4%). Of 588 patients who did not have permanent pacemakers or implantable cardioverter defibrillators, three (0.5%) developed high grade atrioventricular (AV) block (one, Mobitz II AV block; two, third degree AV block). No patients developed torsades de pointes.

Nine of the 572 patients without prior diagnoses of heart failure or cardiomyopathy (2%) were diagnosed with new heart failure or cardiomyopathy. Of the six who underwent echocardiography, four had left ventricular ejection fractions of less than 50%, one was reported as having mild left ventricular impairment but no ejection fraction was recorded, and one had elevated levels of B‐type natriuretic peptide (BNP). Corresponding data were not available for the other three patients, who had clinical diagnoses of heart failure.

Two patients had clinical diagnoses of pericarditis; the troponin level was mildly elevated in one (less than five times the upper limit of normal), suggesting possible myopericarditis. Neither patient underwent echocardiography, cardiac magnetic resonance imaging, or biopsy.

Limitations of our study include the facts that it did not capture subclinical complications, as investigations were undertaken only if clinically indicated, and that there was no comparator group, so we could not compare the incidence of cardiac complications with those of other viral illnesses. Further, we had no information on patient outcomes after their discharge from hospital; cardiac complications such as heart failure and myopericarditis may become apparent only weeks after discharge. Nevertheless, the AUS‐COVID Registry provides important insights into the incidence of clinically apparent cardiac complications in patients hospitalised with COVID‐19.

While clinicians should remain vigilant, the incidence of clinical cardiac complications during index hospitalisations was reassuringly low in our multicentre study of more than 600 consecutive patients admitted to Australian hospitals with COVID‐19.

## Competing interests

No relevant disclosures.

## References

[1] Russo V , Di Maio M , Mottola FF , et al. Clinical characteristics and prognosis of hospitalized COVID‐19 patients with incident sustained tachyarrhythmias: a multicenter observational study. Eur J Clin Invest 2020; 50: e13387. 32813877 10.1111/eci.13387 PMC7460920

[2] Peltzer B , Manocha KK , Ying X , et al. Outcomes and mortality associated with atrial arrhythmias among patients hospitalized with COVID‐19. J Cardiovasc Electrophysiol 2020; 31: 3077–3085. 33017083 10.1111/jce.14770 PMC7675597

[3] Ho JS , Sia CH , Chan MY , et al. Coronavirus‐induced myocarditis: a meta‐summary of cases. Heart Lung 2020; 49: 681–685. 32861884 10.1016/j.hrtlng.2020.08.013 PMC7440036

[4] Arentz M , Yim E , Klaff L , et al. Characteristics and outcomes of 21 critically ill patients with COVID‐19 in Washington State. JAMA 2020; 323: 1612–1614. 32191259 10.1001/jama.2020.4326 PMC7082763

[5] Argenziano M , Bruce S , Slater C , et al. Characterization and clinical course of 1000 patients with coronavirus disease 2019 in New York: retrospective case series. BMJ 2020; 369: m1996. 32471884 10.1136/bmj.m1996 PMC7256651

